# A solution to achieve sequencing from SARS-CoV-2 specimens with low viral loads: concatenation of reads from independent reactions

**DOI:** 10.1186/s12985-024-02347-5

**Published:** 2024-05-30

**Authors:** Alba Cerro-Monje, Sergio Buenestado-Serrano, Rosalía Palomino-Cabrera, Andrea Molero-Salinas, Marta Herranz, Roberto Alonso, Pilar Catalán, Patricia Muñoz, Darío García de Viedma, Laura Pérez-Lago, On behalf of the Gregorio Marañón Microbiology-ID COVID 19 Study Group

**Affiliations:** 1 Servicio de Microbiología Clínica y Enfermedades Infecciosas, Hospital General Universitario Gregorio Marañón, Madrid, Spain; 2 Instituto de Investigación Sanitaria Gregorio Marañón (IiSGM), Madrid, Spain; 3 Escuela de Doctorado, Universidad de Alcalá, Alcalá de Henares, Madrid, España; 4 Departamento de Medicina, Universidad Complutense de Madrid, Madrid, Spain; 5 Centro de Investigación Biomédica en Red (CIBER) de Enfermedades Respiratorias - CIBERES, Instituto de Salud Carlos III, Madrid, España

**Keywords:** SARS-CoV-2, WGS, Low viral load, Concatenation, Genomics

## Abstract

**Background:**

During the pandemic, whole genome sequencing was critical to characterize SARS-CoV-2 for surveillance, clinical and therapeutical purposes. However, low viral loads in specimens often led to suboptimal sequencing, making lineage assignment and phylogenetic analysis difficult. We propose an alternative approach to sequencing these specimens that involves sequencing in triplicate and concatenation of the reads obtained using bioinformatics. This proposal is based on the hypothesis that the uncovered regions in each replicate differ and that concatenation would compensate for these gaps and recover a larger percentage of the sequenced genome.

**Results:**

Whole genome sequencing was performed in triplicate on 30 samples with Ct > 32 and the benefit of replicate read concatenation was assessed. After concatenation: i) 28% of samples reached the standard quality coverage threshold (> 90% genome covered > 30x); ii) 39% of samples did not reach the coverage quality thresholds but coverage improved by more than 40%; and iii) SARS-CoV-2 lineage assignment was possible in 68.7% of samples where it had been impaired.

**Conclusions:**

Concatenation of reads from replicate sequencing reactions provides a simple way to access hidden information in the large proportion of SARS-CoV-2-positive specimens eliminated from analysis in standard sequencing schemes. This approach will enhance our potential to rule out involvement in outbreaks, to characterize reinfections and to identify lineages of concern for surveillance or therapeutical purposes.

**Supplementary Information:**

The online version contains supplementary material available at 10.1186/s12985-024-02347-5.

## Background

On March 11, 2020, the outbreak of SARS-CoV-2 was declared a global pandemic. Since then, the virus has spread to all regions of the planet, creating an unprecedented challenge for researchers and governments. As of February 6, 2023, there have been more than 750 million confirmed cases and more than 6.8 million deaths across all continents [1].

Today, genomic sequencing is a key surveillance tool for understanding the dynamics and spread of the virus and contributes to the implementation of measures to reduce viral spread. At the beginning of the pandemic, whole genome sequencing, using shotgun metagenomics, helped identify and classify SARS-CoV-2 as a new pathogen [2]. Thanks to viral genomic sequencing, it has been possible to design specific primers that have paved the way for targeted amplicon approaches for use in whole genome sequencing that are cheaper and give better results. The ARTIC protocol is the one that has been most adopted for SARS-CoV-2 sequencing [3].

During the 4 years since the pandemic started, an unprecedented effort has been made to sequence the massive number of specimens worldwide, with more than 16.6 million sequences now available from the Global Initiative on Sharing All Influenza Data (GISAID). These NGS data have been key to studying the dynamics of virus spread, the early detection of new and emerging risk variants, the development of vaccines and diagnostic tests such as specific RT-PCR, and essential in the search for specific antiviral strategies [4].

Next-generation sequencing makes it possible to identify mutations with a major impact on severity or transmission capacity, and to identify new variants of concern (VOC) that escape vaccine-generated antibodies or natural infection, are more transmissible, more pathogenic, or have the ability to escape diagnostic detection [5, 6]. NGS is also essential for tracking outbreaks and differentiating between persistent infection and reinfection [7, 8]. Tracking all these factors, as well as variants and their prevalence, is crucial to assess the effectiveness of intervention measures. For this, surveillance is key and NGS is essential for surveillance.

Given the centrality of whole genome sequencing of the virus, a wide variety of sequencing methods have been developed, but all of them face difficulties when it comes to sequencing samples with low viral loads [9–11], mainly because only part of the genome is covered by the reads obtained. World Health Organization guidelines for genomic sequencing suggest that the whole genome can be sequenced in samples with RT-PCR cycle threshold (Ct) values up to 30, whereas only partial genome sequencing can be achieved for Ct values of 30 to 35 [12]. Several papers define RT-PCR Ct thresholds above which sequencing is not even attempted [11, 13].

Specimens with low viral SARS-Cov-2 loads are expected at the beginning or end of infection, as well as in asymptomatic or mildly ill patients who may act as vectors of transmission [9, 14, 15]. Furthermore, extra-respiratory samples, such as plasma and urine, often have lower viral loads [15]; these samples can be useful, and indeed necessary, to study patients with persistent infection or long-term COVID.

In this study, we investigated the potential improvement that may be derived from bioinformatically concatenating reads obtained after performing three independent sequencing reactions on samples with low viral loads. Our aim was to improve the suboptimal results expected from the standard single analysis of these specimens.

## Materials and methods

### Clinical specimens

The study samples were collected from cases diagnosed at the Gregorio Marañón Hospital, Madrid, Spain, between February and May 2022. Diagnosis of COVID-19 was performed by extraction and purification of viral RNA from 300 µL of nasopharyngeal exudate with the KingFisher System (ThermoFisher Scientific, Waltham, MA, USA). Purified RNA was assayed by RT-PCR using the TaqPath COVID-19 CE-IVD RT-PCR kit (ThermoFisher Scientific, Waltham, MA, USA), which targets the open reading frame 1ab (ORF1ab), nucleoprotein (N2), and spike (S) genes. The Ct value for the N2 gene was selected as the reference to infer viral load.

The specimens for the study corresponded to the remains of nasopharyngeal exudate that had been used for diagnostic purposes, then stored at -70 °C. The study was performed on 30 samples: 24 were samples with Ct > 32 (R-1 to R-24) and the other 6 (R-25 to R-30) were the result of diluting samples, which had a lower Ct value, to achieve a Ct > 32. The Ct of these final dilutions was tested by RT-PCR.

### Standard whole genome sequencing

Sixteen µL of RNA was used as template for reverse transcription with the LunaScript® RT SuperMix Kit (New England Biolabs, Ipswich, MA, USA). Whole-genome amplification of SARS-CoV-2 was performed with an Artic V4.1 NCov-2019 panel of primers (Integrated DNA Technologies, Inc., Coralville, IA, USA; artic.networkncov-2019) and Q5 Hot Start DNA polymerase (New England Biolabs, Ipswich, MA, USA). Libraries were prepared with the Illumina DNA Prep kit (Illumina lnc., CA, USA) using the Sciclone G3 NGSX IQ workstation (PerkinElmer, Waltham, MA, USA). They were then quantified with the Quantus fluorometer (Promega, WI, USA), pooled at equimolar concentrations (4nM), prior to sequencing in pools on the Miseq platform (Illumina Inc, CA, USA).

**Bioinformatics analysis.** An in-house analysis pipeline was applied (https://github.com/MG-IiSGM/covid_multianalysis). Briefly, the pipeline goes through the following steps: (1) pre-processing and quality assessment for FastQ files, using FastQC v0.11.933 for quality control and fastp v0.20.134 to trim adapters and low quality reads; (2) mapping reads to the reference genome with the Burrows-Wheeler Aligner (BWA-MEM) 0.7.17-r1188.35; (3) marking and removing PCR duplicates with Picard v2.23.4,36; (4) variant calling with iVAR v1.2.337, using the Wuhan-1 sequence (NC_045512.2) with the following parameters: quality (-q 20), frequency (-t 0.8) and depth (-m 20); 4) creating the consensus genome with iVAR v1.2.337, using the same parameters, except for depth, which was increased to 30; and (5) predicting lineage with Pangolin v4.1.3.

### Concatenated sequence analysis

Each specimen was sequenced three times independently from the same extraction, following the standard procedure. The result of each sequencing experiment was designated a replicate, and each replicate was labelled A, B or C. FastQ files of replicates were concatenated by an automated script in Linux Bash terminal, using the “cat Isolate1 Isolaten > output” command to group the reads from all replicates into the same single FastQ file. The results of concatenating two replicates were designated AB, AC, BC, and the concatenation of three replicates was designated ABC.

To compare the results between standard single sequencing and the concatenation alternative, one of the replicates was randomly selected as reference, and the replicates were then considered as second and third replicates.

## Results and discussion

### Determination of a Ct threshold associated with optimal sequencing

The decision of whether to exploit a SARS-CoV-2 sequence for further analysis depends primarily on the percentage and breadth of genome coverage by sequencing reads. The most general requirement is genomic coverage of > 90% at > 30x depth.

We first evaluated whether a threshold could be found for the Ct value obtained in RT-PCR testing of a specimen, in order to determine whether there was an increased probability of obtaining suboptimal sequencing data below that threshold. For this analysis, we used the data obtained from the 7253 SARS-CoV-2 specimens sequenced in our laboratory from the beginning of the pandemic until March 2022.

**Fig. 1:**
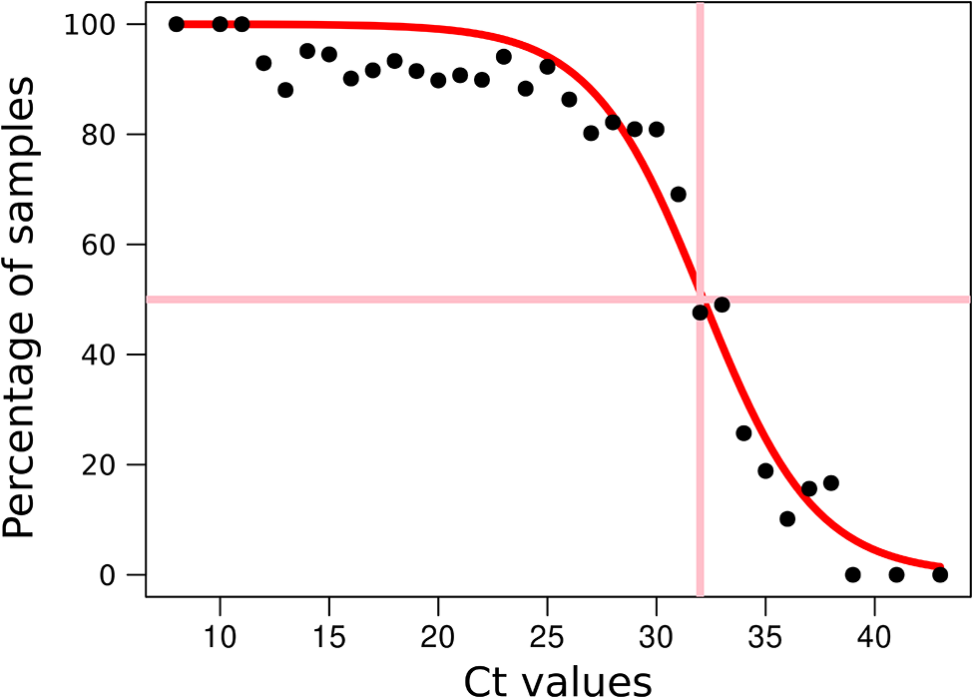
Proportion of samples with optimal coverages (> 90% of genomic coverage at > 30X), distributed according to the intervals of consecutive Ct values (9–10, 10–11, 11–12, etc.). Each dot corresponds to the proportion of samples with optimal genomic coverages for each consecutive Ct interval

The correlation between the Ct value (N2 gene) and the proportion of specimens that gave sequencing coverage values above the quality thresholds was analysed. A reverse sigmoid relationship was found between these two parameters (Fig. 1). Using the nonlinear least squares (nls) function in R, we fitted the data to a reverse sigmoid function, as shown in the equation, where P is the proportion of samples (with Ct within the interval [Ct_1_, Ct_2_⌉, where Ct_1_ and Ct_2_ are all the possible intervals between consecutive Ct values, i.e. 22–23, 23–24, 24–25), with genomic coverage > 90% at > 30x depth, and Ct′ = Ct_1_, obtaining the values a = -0.4 and b = 32.14.

$$\text{E}\text{q}: P=1-\frac{1}{\begin{array}{c}1+{e}^{a(C{t}^{{\prime }}-b)}\end{array}} \qquad P=1-\frac{1}{\begin{array}{c}1+{e}^{-0.4(C{t}^{{\prime }}-32.14)}\end{array}}$$

From Eq, we deduced that the Ct value at which half the samples sequenced (*P* = 0.5) showed genomic coverage > 90% > 30x was 32.14, which was considered the threshold for predicting an optimal or suboptimal sequencing result.

**Fig. 2:**
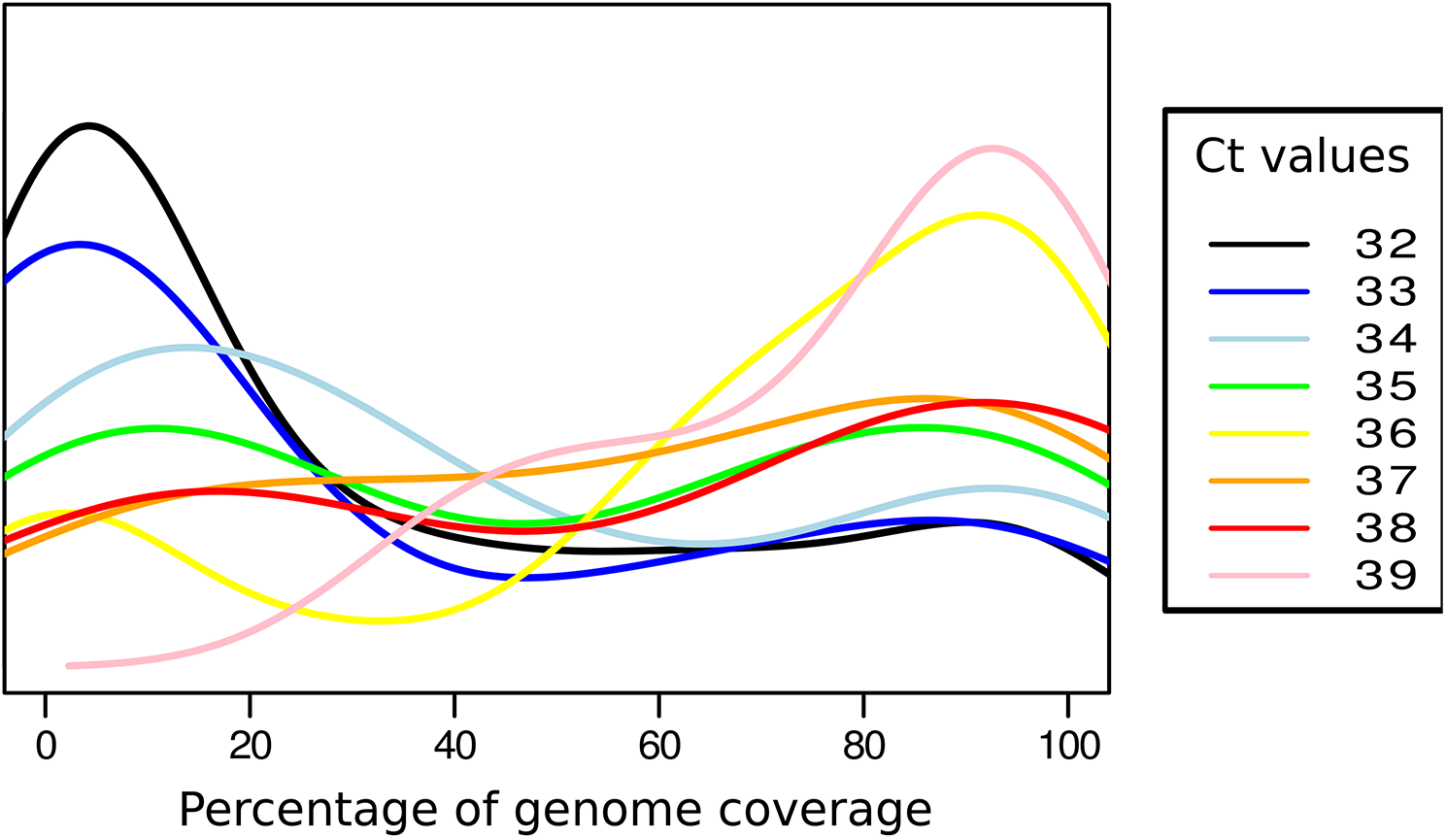
Distribution of the percentage of genome coverage (0-100%) for the samples belonging to a selection of Ct values (from Ct 32 to Ct 39; *n* = 524). Each line corresponds to the behaviour of the samples sharing one of those Ct values

Further analysis of specimens with Ct values > 32 indicated that, also in the subgroup of samples with low viral load, the proportion of sequences giving good genomic coverage continued to be dependent on viral load (Fig. 2).

The frequency of specimens with viral load above the Ct > 32 threshold is sufficiently high to support procedures that allow sequencing them. At our institution, 542 of all sequenced specimens (7.5%) had Ct > 32, and this percentage increased significantly during the pandemic to reach 40% of all new COVID-19 diagnoses in April 2022. For a more detailed understanding of the consequences of not obtaining an optimal sequencing result for these specimens during the pandemic, we reviewed the type of analytical request for which they were needed: outbreak characterization (9.8%), breakthrough infections (5.69%), characterization of recurrences (17.4%), healthcare worker infections (16.77%), lineage assignment in recently arrived international travellers (6.64%) and general requests for lineage assignment (12.65%).

### Concatenation of reads from triplicate sequencing reactions

For specimens with low viral loads leading to suboptimal sequencing coverage, we hypothesized that the uncovered regions in the genome would be random and therefore different in independent sequencing reactions of the same specimen. Based on this assumption, we evaluated whether concatenation of the reads obtained from three independent sequencing reactions could compensate for the regions not covered in each independent replicate, ultimately providing adequate global coverage. Similar efforts have not been undertaken or evaluated before and have only been suggested as a potential solution to overcome the limitations of sequencing specimens with low viral loads [8].

#### Quality of reads obtained

Thirty samples with Ct > 32 were selected for sequencing in triplicate. The genomic coverages obtained in the 90 replicated sequences were varied and therefore unpredictable. While some specimens offered optimal results in all replicates (R-6, R-8, R-21 and R-24), others failed to reach the quality threshold in any of their 3 replicates, with coverages lower than 8% in all replicates (R-15 and R-16; Supplementary Table 1). We distinguished between specimens with reproducible results, those where the standard deviation of replicates fell within 25% of the mean, and those that were non-reproducible. Half of the specimens gave non-reproducible results (Supplementary Table 1). The distribution between reproducible and non-reproducible results was not associated with the Ct values of the specimens.

#### Improvements as a result of replicate read concatenation

To determine whether a progressive improvement was obtained by concatenating reads from one additional replicate or from two, we randomly selected one of the three replicates to be used as reference; the other two were then used as first and second providers of new reads to be concatenated with the reference.

As criteria to evaluate whether replicate read concatenation improves the sequencing results, we defined two quantitative targets (achieve the standard coverage threshold, or improve them even if the threshold is not reached) and one qualitative target (number of samples where SARS-CoV-2 lineage can be assigned).

##### Specimens reaching the coverage threshold

In four specimens (R-6, R-8, R-21 and R-24), all the three replicates reached the optimal quality thresholds, and in another specimen (R-19), the replicate randomly selected as a reference also exceeded the required threshold and so could not be used to evaluate improvements resulting from read concatenation (Supplementary Table 1). All five specimens had a Ct value of 34, close to the threshold of 32, which might explain their results.

On the other hand, seven of the specimens (28%) with suboptimal results exceeded the coverage threshold after concatenation of replicate reads. In six of these, it was sufficient to concatenate just two replicates, while in the remaining one (R-7), it was necessary to concatenate all three (Table 1). The remaining specimens did not reach the coverage threshold even after concatenating all three replicate reads (Supplementary Table 1).

**Table 1:** Percentage of the genome covered (> 30X) in the three independent replicates, concatenating two replicates (2X) and concatenating three replicates (3X) in the specimens that overcame the quality threshold when concatenating. The replicates which were randomly selected as references are shaded

Sample ID	Ct	1X			2X	3X
		A	B	C		
R-3	38.3	**87.63**	72.65	94.28	95.2 (AB)	99.02
R-5	33	90.28	68.06	**87.11**	97.47 (AC)	98.35
R-7	33.3	67.06	**51.97**	69.81	84.56 (BC)	92.22
R-9	35	83.62	89.34	**89.15**	97.59 (BC)	98.51
R-20	34	80.41	**85.46**	83.84	92.17 (BC)	94.27
R-28	34	96.52	95.79	**75.63**	98.69 (BC)	99.59
R-29	33	78.67	76.65	**72.73**	94.04 (AC)	96.4

For specimens that reached the coverage threshold after read concatenation, two patterns were distinguished: (i) those where some of the replicate reads reached the coverage threshold before concatenation (R-3, R-5 and R-28) or were very close to it (> 85% >30x; R-9 and R-20), and (ii) those that exceeded the threshold after concatenation despite suboptimal coverage (< 80%) of the reads from all replicates (R-7 and R-29; Table 1). It is worth mentioning that the coverage threshold was reached even in one sample (R-7) with clear suboptimal (52%, 67.1% and 69.8%) coverage in all three replicates. These findings support our assumption that the concatenation of reads compensates for the different regions with suboptimal coverage found in independent replicates of the same specimen.

##### Specimens improving coverage

For the eighteen cases that did not achieve the quality coverage threshold after concatenation of replicates, it was still of interest to quantify the magnitude of improvement achieved, as expressed by the additional percentage of genome coverage achieved by concatenation of either two or three replicates.

Concatenation of two replicates recovered on average an additional 19.8% of the genome (standard deviation = 19.5) as compared to the values obtained in the single, randomly selected reference reaction (Table 2). When all three replicates were concatenated, an additional 31.7% of the genome was recovered (standard deviation = 17.06). The large standard deviations are due to the wide variation in coverage provided by the different replicates. We also considered what the improvement achieved by concatenating two and three replicates would have been if we had compared with the replicate offering the worst results instead of the random reference (Table 2), and a further 4% improvement in genome coverage would have been obtained in both cases (Table 2).

**Table 2:** Percentage of genome covered (> 30X) in the three independent replicates, concatenating two replicates (2X) and three replicates (3X) in the specimens that did not exceed the quality threshold when concatenating. Recovery rates of specimens concatenating two replicates and three replicates when a random replicate was selected and when the worst replicate was selected. The replicates which were randomly selected as references are shaded

	Ct	1X			2X		3X	2X − 1X	3X − 1X	2X − 1X	3X − 1X
		A	B	C				Improvement	Improvement	Worst replicate improvement	Worst replicate improvement
R-1	36	**28.11**	29.19	14.52	44.26	(AB)	51.21	16.15	23.10	29.74	36.69
R-2	36	**10.20**	45.77	1.24	11.20	(AC)	50.39	1.00	40.19	9.96	49.15
R-4	38.1	11.30	**22.68**	29.51	31.19	(AB)	49.96	8.51	27.28	19.89	38.66
R-10	36	42.28	22.36	**27.30**	56.29	(AC)	61.85	28.99	34.55	33.93	39.49
R-11	35	40.99	**6.57**	2.33	8.93	(BC)	49.56	2.36	42.99	6.60	47.23
R-12	34	66.12	**0**	0	66.19	(AB)	66.21	66.19	66.21	66.19	66.21
R-13	37	7.94	**2.64**	32.17	36.37	(BC)	47.72	33.73	45.08	33.73	45.08
R-14	35	42.91	14.42	**22.82**	34.02	(BC)	65.27	11.20	42.45	19.60	50.85
R-15	35.4	7.67	0.93	**1.35**	2.19	(BC)	9.80	0.84	8.45	1.26	8.87
R-16	34	3.63	**4.57**	5.94	10.47	(BC)	14.18	5.90	9.61	6.84	10.55
R-17	34	55.89	52.21	**53.99**	68.54	(BC)	82.80	14.55	28.81	16.33	30.59
R-18	36	1.72	2.14	**24.35**	26.52	(AC)	27.71	2.17	3.36	24.80	25.99
R-22	35	66.17	**66.65**	66.15	74.65	(BC)	81.28	8.00	14.63	8.50	15.13
R-23	36	14.19	65.26	**20.40**	65.61	(BC)	65.89	45.21	45.49	51.42	51.70
R-25	34	**1.39**	54.45	5.40	55.54	(AB)	57.71	54.15	56.32	54.15	56.32
R-26	35	47.91	**35.44**	32.78	68.35	(AB)	74.57	32.91	39.13	35.57	41.79
R-27	33	15.02	**0**	7.78	7.78	(BC)	21.60	7.78	21.60	7.78	21.60
R-30	36	**6.78**	19.69	7.77	23.72	(AB)	29.69	16.94	22.91	16.94	22.91
MEAN								19.81	31.79	24.62	36.60
STDEV								19.53	17.06	18.40	16.36

The markedly suboptimal coverages obtained from the reference replicates in these samples (18.6% and 13.8% on average for the randomly selected reference and the worst replicate, respectively; Table 2) explains why they did not reach the coverage threshold despite the reasonable improvement in average coverage provided by the concatenated replicates. Unlike the results obtained previously in specimens that did reach the coverage thresholds, where concatenation of two replicates was sufficient to make an improvement, in this case, concatenation of all three replicates resulted in a significant improvement in values relative to those obtained by concatenating just two. This would be due to the lower initial coverage, and thus the greater opportunities for improvement shared by the latter, more suboptimal specimens.

As expected, when coverage of the randomly selected reference replicate in the comparison was notably suboptimal, the maximum improvements were recorded after concatenation. For example, the reference replicate of specimen R-11 showed 6.6% coverage, which increased to 49.6% after concatenation (Table 2). Due to the wide variation in coverage among replicates, the improvements were obviously greater when the replicates behaved more consistently with each other, as in sample R-17, where each replicate showed a coverage of around 55%, which improved to 82.8% after concatenation of all three replicates (Table 2).

In most of the cases where the replicates showed coverage variance, the progressive improvement associated with concatenation corresponded mostly to the sum of the coverages provided by the replicates (Table 2 and Supplementary Table 1). This again supports our view that uncovered regions in suboptimal sequencing reactions differ from replicate to replicate and that concatenation of reads from independent sequencing reactions progressively closes the different read gaps in the genome. By way of contrast, in certain specimens (R-23), we did not observe this behaviour, and the final coverage after concatenation did not correspond to the sum of the coverages of the independent replicates. This could mean that some region of the genome in the specimen was not represented, or was degraded, and that concatenation was not able, therefore, to provide the expected improvements.

In summary, an improvement >40% in genome coverage (in the 39% of cases that did not pass the quality criterion) would justify using the concatenation strategy, even assuming that only specimens with not-too-suboptimal coverage values reached the quality threshold after concatenation.

##### SARS-CoV-2 lineage assignment capability

One of the main goals of SARS-CoV-2 sequencing in the surveillance of sequentially emerging variants is to assign lineage. Therefore, in addition to evaluating the specific quantitative improvement in coverage achieved after concatenation, we also determined the qualitative approach to lineage assignment in specimens where this was initially not possible.

In 14 specimens (46%), lineage was assigned from the sequence obtained in each replicate (Supplementary Table 1) and so could not be used to assess improvement. The minimum coverage obtained in a replicate for these specimens was 51.2% (Supplementary Table 1).

In specimens where lineage could not be assigned from sequences obtained in a single replicate, concatenation enabled lineage assignment in 11 (68.7%) after concatenation of two or three replicates (6 and 5 cases, respectively; Table 3). Lineage assignment after concatenation was also possible in specimens where sequence coverages for all three replicates were below 30% (cases R1 and R4; Table 3). None of these eleven samples reached the quality coverage threshold after concatenation (Table 3). Lineage assignment only requires that the genomic positions of the markers be well covered, which means that it can be performed even when much of the remainder of the genome is not properly covered. This implies that any degree of improvement in coverage provided by concatenation could be relevant to allow lineage assignment, even without reaching the coverage quality threshold after concatenation.

**Table 3:** Percentage of the genome covered (> 30X) in the three independent replicates, concatenating two replicates (2X) and three replicates (3X) in the specimens whose lineage could not be identified with one replicate, but could be assigned after concatenating two or three replicates. The replicates which were randomly selected as references are shaded

Sample (ID)	Ct	1X			2X		3X	Lineage		
		A	B	C				1X	2X	3X
R-1	36	**28.11**	29.19	14.52	44.26	(AB)	51.21	Unassigned	BA.1.1.1	BA.1
R-2	36	**10.20**	45.77	1.24	11.20	(AC)	50.39	Unassigned	Unassigned	BA.1.17.2
R-4	38.1	11.30	**22.68**	29.51	31.19	(AB)	49.96	Unassigned	Unassigned	BA.1.17
R-10	36	42.28	22.36	**27.30**	56.29	(AC)	61.85	Unassigned	BA.2	BA.2
R-11	35	40.99	**6.57**	2.33	8.93	(BC)	49.56	Unassigned	Unassigned	BA.2
R-12	34	66.12	**0**	0	66.19	(AB)	66.21	Unassigned	BA.2	BA.2
R-13	37	7.94	**2.64**	32.17	36.37	(BC)	47.72	Unassigned	Unassigned	BA.2
R-14	35	42.91	14.42	**22.82**	34.02	(BC)	65.27	Unassigned	Unassigned	BA.2
R-23	36	14.19	65.26	**20.40**	65.61	(BC)	65.89	Unassigned	BA.2	BA.2
R-25	34	**1.40**	54.45	5.40	55.54	(AB)	57.71	Unassigned	AY.43	AY.43
R-26	35	47.91	**35.44**	32.78	68.4	(AB)	74.57	Unassigned	BA.1.1.2	BA.1.1.2

Lineage could not be assigned in only 5 specimens (16.1%), even after three replicates were concatenated (Supplementary Table 1). The coverages reached for these specimens after concatenation of all three replicates ranged between 9.8% and 29.7% (Supplementary Table 1).

## Conclusions

Despite the optimal results obtained from a proportion of specimens with low SARS-CoV-2 loads (Ct > 32), the strategy proposed in our study would be of benefit to improve the unpredictable quality of their sequencing data. By bioinformatically concatenating the reads obtained from three independent sequencing reactions of the same specimen, we successfully reached the coverage quality thresholds (genomic coverage > 90% >30x) required to perform a complete sequencing analysis on specimens where it would otherwise have been impaired. Furthermore, even in specimens where optimal coverage was not achieved, a significant increase in the percentage of genome coverage was achieved after read concatenation. Finally, SARS-CoV-2 lineage was assigned in the majority of specimens where it was missing. Given that a notable proportion of specimens have a low viral load, concatenation of replicates offers a possible solution to rule out outbreak involvement, to assess reinfections, or assign lineage for surveillance or therapeutical purposes, among other things [7, 8, 16, 17]. We would recommend to perform three sequencing replicates for a single extraction of the same specimen, for those specimens with Ct > 32 which fail to achieve enough coverage after a first sequencing round. Our proposal will allow the extraction of valuable information that lies untapped in specimens with low viral load with the current standard sequencing schemes.

### Electronic supplementary material

Below is the link to the electronic supplementary material.

Supplementary Material 1

## Data Availability

The data supporting the findings of this study (FastQ files) were deposited at ENA (https://www.ebi.ac.uk) under the project accession number PRJEB60934.
